# 亚肺叶切除治疗老年Ⅰ期非小细胞肺癌进展

**DOI:** 10.3779/j.issn.1009-3419.2017.10.08

**Published:** 2017-10-20

**Authors:** 思远 董, 林 张

**Affiliations:** 110001 沈阳，中国医科大学附属第一医院胸外科 Department of Toracic Surgery, the First Hospital of China Medical University, Shenyang 110001, China

**Keywords:** 亚肺叶切除, Ⅰ期, 肺肿瘤, 老年患者, Sublobectomy, Stage Ⅰ, Lung neoplasms, Elderly

## Abstract

肺癌是世界范围内发病率最高的恶性肿瘤，且有逐年增加的趋势，随着人口老龄化和薄层电子计算机断层扫描（computed tomography, CT）的应用，老年早期肺癌被越来越多的发现，手术仍然是这类人群的主要治疗方式，目前主要手术方式为肺叶切除和亚肺叶切除两种，由于老年群体的特殊性，对手术方式的选择也趋于“个性化”，本文对这两种手术方式的选择问题做一综述。

## Abstract (en)

Lung cancer continues to be one of the leading causes of cancer-related mortality worldwide and its incidence is still increasing. With aging population and development in imaging technology, many more elderly with early-stage lung cancers are being diagnosed than in the past. Lobectomy and sub-lobar resection are two main surgical approach for the treatment for this kind of patients. This review aimed to compare these two methods for this hot issue.

非小细胞肺癌（non-small cell lung cancer, NSCLC）目前仍然是世界范围内发病率最高的恶性肿瘤，且发病率有上升的趋势^[1]^。统计数据表明70岁为NSCLC的发病高峰^[2]^，随着人口老龄化的到来和高分辨率电子计算机体层扫描（computed tomography, CT）及正电子发射计算机断层显像（positron emission tomography-CT, PET-CT）的广泛应用，越来越多的老年早期肺癌被发现，而且我们相信未来几十年此类患者的数量会进一步增加。手术仍然是治疗早期NSCLC的主要手段，有研究^[3, 4]^发现对于难以耐受肺叶切除的老年早期肺癌患者亚肺叶切除能够获得与肺叶切除相同的手术效果，但对于能够耐受肺叶切除的老年早期患者是否应进行亚肺叶切除尚有争议。现就老年（> 70岁）Ⅰ期NSCLC手术方式的选择及相关问题做一综述。

## 1 肺叶切除还是亚肺叶切除？

Ginsberg等^[5]^于1995年报道了一组纳入247例肺叶切除和亚肺叶切除（肺段切除术及肺楔状切除术）治疗T1N0期NSCLC的前瞻性、随机性的Ⅲ期临床研究，结果显示：亚肺叶切除术较肺叶切除术后复发率增加75%，总死亡率及肺癌相关死亡率分别增加30%及50%，且进行亚肺叶切除不能减少术后并发症和围手术期死亡率的发生，也不能保留更多的肺功能。2017年美国国立综合癌症网络（National Comprehensive Cancer Network, NCCN）指南^[6]^和2015年的美国胸部医师协会（American College of Chest Physicians, ACCP）指南^[7]^也同样遵循该原则。解剖性肺叶切除加系统性淋巴结廓清多年来一直为NSCLC的标准术式，亚肺叶切除仅仅用于治疗无法耐受肺叶切除的患者。但该项研究距今已超过20年，无论是手术技术还是相关的检查手段都有了较大的变化，尤其是PET-CT的应用能够更准确的对肺部病灶进行较准确术前分期。如Yendamuri^[8]^通过对1987年-2008年Surveillance Epidemiology End Results（SEER）数据库中直径≤2 cm的NSCLC进行分析发现：1988年-1998年亚肺叶切除的生存率和无病生存率均劣于肺叶切除；1999年-2004年肺楔状切除仍然劣于肺叶切除，但肺段切除同肺叶切除获得了类似的生存率；2005年-2008年时间段的亚肺叶切除上述二项指标与肺叶切除无差异。故有许多机构对早期肺癌实施较小范围的切除，尤其是解剖性肺段切除^[9]^。Schuchert^[10]^的回顾性研究、Cao^[11]^和Nakamura^[12]^的荟萃分析均表明：对于Ⅰ期患者亚肺叶切除能够获得与肺叶切除相同的效果。

由于老年群体的特殊性，许多 > 70岁的老年人同时合并慢阻肺、糖尿病及心脑血管病等慢性疾病，对抗手术风险能力显著低于年轻患者，且预期生存期亦低于年轻患者。如针对这些老年患者仍然应用来自年轻患者的研究结果，可能存在过度治疗，并可能造成术后并发症甚至病死率的增加。来自美国梅奥诊所的数据表明：老年患者（≥80岁）肺叶切除术并发症发生率为48%，死亡率为6.3%^[13]^，二者均显著高于年轻患者。对于Ⅰ期老年NSCLC患者能否从创伤较大的根治性肺叶切除术中获益更大仍然存在分歧^[14, 15]^。2005年Mery等^[16]^通过回顾性SEER数据库14, 555例Ⅰ期及Ⅱ期NSCLC发现：在71岁以上人群中，肺叶切除和亚肺叶切除在远期生存率上无差异。2016年Razi等^[17]^再次利用SEER数据库对 > 75岁的T1aN0M0患者进行了分析，结果显示对于此类患者无论行肺楔状切除、肺段切除还是肺叶切除对生存率均无影响。同样的结论还来自匹兹堡大学研究中心^[18]^：在 > 75岁的Ⅰ期NSCLC患者中，亚肺叶切除能够获得与肺叶切除类似的生存率，且术后并发症的发生率更低。

对于手术风险相对较高的老年患者，“妥协性”的亚肺叶切除能够在一定程度上降低手术风险。早在1999年Takizawa等^[19]^就通过比较40例亚肺叶切除和40例肺叶切除患者12个月后肺功能发现：进行亚肺叶切除术较肺叶切除术能够更好的保护残余肺功能，尤其是FEV_1_。过去10年间国际上共有30余篇论文报道了肺叶切除与亚肺叶切除治疗早期NSCLC的效果比较，其中7篇研究对象为年龄 > 70岁的老年患者^[14, 20-25]^，这7项研究均得出了亚肺叶切除能够获得和肺叶切除类似的1年、3年和5年生存率，但这7篇论文均为回顾性研究，研究结果的证据级别偏低。

主张对老年患者进行亚肺叶切除的研究者认为，在完整切除肿瘤基础上，最大限度的保留有功能的肺组织能够减少围手术期并发症的发生。反对亚肺叶切除的学者认为，虽然这些结果显示了亚肺叶切除和肺叶切除获得相同的效果，但这些研究均为回顾性研究，结论的证据级别较低；且研究中并没有说明这些进行亚肺叶切除的患者是“意向性”亚肺叶切除，还是因为高龄、心肺功能差以及其他合并症，而难以耐受肺叶切除而行的“妥协性”亚肺叶切除，故存在显著的选择偏倚。

我国王俊教授团队于2015年开展了针对 > 70岁的cT1N0M0的NSCLC患者亚肺叶切除与肺叶切除的前瞻性临床随机对照实验研究（STEPS研究）^[26, 27]^，希望此项研究能够为这个争议问题带来有指导意义的临床证据。

## 2 楔状切除还是解剖性肺段切除？

亚肺叶切除包括解剖性肺段切除和非解剖性楔状切除，两种方式各有优劣，对亚肺叶切除具体方式的选择仍然是一个争议的话题。解剖性肺段切除能够依据解剖结构将肺癌引流区域的肺组织完整切除，并同时进行一定的淋巴结采样，但缺点是手术较为复杂，对技术要求较高，肺段的界限较难精准确定，且术后并发症的发生率高于楔型切除。肺楔状切除对手术技术的要求相对较低，且手术创伤小、时间短以及术后并发症发生率低，对于老年患者优势明显，但总体生存率低于肺段切除。

2013年Yendamuri等^[8]^回顾性分析了3, 525例来自SEER数据库Ia期NSCLC生存资料，结果显示肺段切除在生存率方面显著优于楔状切除，且在年龄 > 70岁的患者中这种优势依然存在。2014年Reveliotis等^[28]^通过回顾性分析45篇文章对这一问题做了综述，认为肺段切除能够获得更好的局部控制率和远期生存率，建议优先选择肺段切除治疗早期肺癌。2016年Hou等^[29]^的荟萃分析表明：对于≤2 cm的肿瘤患者，楔状切除能获得与肺段切除相同的效果，但对于肿瘤直径 > 2 cm患者，肺段切除优势明显。

然而，Smith等^[30]^通过回顾性分析了1, 568例肺楔状切除和378例行肺段切除的Ia期NSCLC病例发现：肺段切除生存率显著高于行肺楔状切除的患者，但这种优势在年龄 > 70岁的人群中并未显现。2012年来自美国匹兹堡医学中心的数据表明：对于Ia期NSCLC患者，肺段切除和楔状切除在5年生存率上无差别^[31]^。Tsutani等^[32]^的一项前瞻性研究比较了56例行肺段切除和93例肺楔状切除的Ⅰ期肺腺癌病例，两组在无病生存率和总体生存率上并无差异。但这项研究中对于Ia期患者更多的采取了楔状切除术，而Ib期的患者更多的采用了肺段切除的方式，故此项研究可能存在潜在的选择性偏倚。2016年Altorki等^[33]^通过回顾性分析289例cT1N0 NSCLC患者，虽然解剖性肺段切除患者进行了更多的淋巴结清扫，但生存率上二者并无差异。

肺段切除与楔状切除的远期效果比较尚存争议，总体而言，目前更多的研究结果支持肺段切除术治疗早期肺癌疗效优于楔形切除。上述结果也均来自没有进行年龄分层的研究，目前还没有专门针对70岁以上的Ⅰ期患者进行的前瞻性研究。

## 3 影响亚肺叶切除效果的因素（肿瘤位置、直径、切缘与淋巴结）

### 3.1 肿瘤位置

对亚肺叶切除适应症的把握是首先要解决的问题，主要涉及到肿瘤位置、肿瘤直径大小、切缘距离及是否清除淋巴结，下面分别就上述问题进行阐述。Sienel于2007年回顾性分析了49例行肺段切除的T1N0M0肺癌患者，研究发现切缘病理阳性、病灶累及胸膜和淋巴管阳性为复发的高危因素；研究还发现肺段切除部位对复发亦有影响，其中肺段切除S7-10（0/6）和S4-5（0/5）无复发，S6复发1例（1/8），S1-3复发率较高（7/30），建议若肿瘤位于S1-3肺段，则尽量避免行肺段切除^[34]^。2016年Luzzi等^[35]^通过回顾性分析了279例直径 < 3 cm的周边型肺腺癌病例，再次证实了胸膜受累为肿瘤复发的高危因素，若术中证实胸膜受累应避免进行亚肺叶切除术。

### 3.2 肿瘤直径

肿瘤大小是影响肿瘤术后是否复发的重要因素之一，目前亚肺叶切除主要适用于Ia期以及心肺功能差不能耐受肺叶切除的患者，Nomori等^[36]^回顾性分析了179例行肺段切除的周围型T1N0M0 NSCLC患者，其中134例直径≤2 cm，45例直径2.1 cm-3 cm，两组5年生存率分别为94%和81%，具有统计学差异。Okada等^[37]^的研究也发现对于2 cm-3 cm的NSCLC，在远期生存率方面肺叶切除优势明显。上述结果表明亚肺叶切除更适合肿瘤直径 < 2 cm的病例，对于直径 > 2 cm的肺癌选择进行亚肺叶切除需慎重。随着薄层CT的应用，越来越多的磨玻璃结节（ground-glass opacity, GGO）被发现，其病理类型多为非典型腺瘤增生、原位癌及微浸润癌。进行亚肺叶切除可获得良好的效果，Sugi等^[38]^研究发现当肿物直径 < 1.5 cm且GGO成分 > 75%时，行楔状切除5年无病生存率可达到100%；肿物直径1.5 cm-2.0 cm且以GGO成分为主时，行肺段切除5年无病生产率可达到90.5%。2017年Qiu等^[39]^的研究也证实了对于肿物直径 < 2 cm的病灶，肺叶和亚肺叶切除能够获得相同的肿瘤根治效果，故肿物大小及GGO成分的比例可能是决定是否适合进行亚肺叶切除的重要决定因素。

### 3.3 是否清除淋巴结

对于早期肺癌是否行淋巴结采样或廓清是一个有争议的问题，2017年Stiles等^[40]^及其团队对楔状切除是否行淋巴结清除进行了研究：共计196例行楔状切除的术前分期为Ia期的肺癌患者，138例进行了淋巴结清除（平均清除4组），其中有6例为淋巴结转移癌，58例没有进行淋巴结清除。两组在手术时间、失血量、引流管留置时间及住院时间无差别，但行淋巴结清除组的复发率显著低于单纯行楔状切除组。2013年Smith等^[30]^的研究认为在行解剖性肺段切除时，应该对引流区及肺门的淋巴结进行采样，若术中冰冻病理为转移，则应放弃肺段切除，改为根治性肺叶切除。

### 3.4 切缘距离

亚肺叶的切除距离的选择也是一个有争议的问题，越来越多的证据表明切缘距离是影响手术效果的关键。最早的研究来自EI-Sherif团队^[41]^，他们比较了40例切缘 > 1 cm和41例切缘 < 1 cm的进行亚肺叶切除（包括楔状和肺段）的早期NSCLC复发情况，其中切缘 > 1 cm的复发率（3/40）显著低于切缘 < 1 cm（6/41）的复发率。第六版NCCN指南指出亚肺叶切除的肺残缘，需要距离肿瘤组织2 cm以上或大于病灶最大直径，并推荐用于①不能耐受肺叶切除患者；②最大直径≤2 cm的周边病灶，且至少具备病理类型为原位癌、GGO成分 > 50%或肿瘤倍增时间 > 400 d特征中的一项。Mohiuddin等^[42]^的研究结果支持了指南的结论，他们的研究共纳入了479例病灶直径 < 2 cm的患者，结果显示切缘距离1 cm的局部复发率显著低于切缘0.5 cm的患者，但切缘 > 1.5 cm后并不能获得额外收益。然而，2015年Maurizi等^[43]^将182例Ⅰ期肺癌行肺楔状切除的患者根据切缘距离分为三组： < 1 cm（30例）、1 cm-2 cm（80例）及 > 2 cm（72例），无论是局部复发率、远隔转移率还是总体生存率三组均无差异。

## 4 总结

随着临床诊断及手术水平的提高，肺癌的治疗既需要“规范化”，也需要“个体化”。鉴于老年患者自身条件的特殊性且预期寿命有限，相对创伤较小的亚肺叶切除可能获得与肺叶切除相同的效果，亚肺叶切除适应症的把握、切缘的距离及淋巴结清扫范围的规范化是亟需解决的问题。通过总结文献我们发现满足以下条件老年患者可试行亚肺叶切除：①肿瘤直径 < 2 cm的周边病灶，并确保切缘 > 2 cm，必要时行术中病理确保切缘阴性；②术前CT显示GGO成分大于50%；③术中应进行淋巴结采样，确定无阳性淋巴结；④肿瘤若位于S1-3肺段则应尽量避免进行亚肺叶切除。

目前关于支持亚肺叶切除治疗老年早期肺癌的证据主要来自回顾性研究，相对创伤较小的亚肺叶切除能否成为老年早期NSCLC患者的标准术式，尚需要大规模的随机对照实验证明。

## References

[1] Siegel RL, Fedewa SA, Miller KD (2015). Cancer statistics for Hispanics/Latinos, 2015. CA Cancer J Clin.

[2] Yancik R (2005). Population aging and cancer: a cross-national concern. Cancer J.

[3] Okumura M, Goto M, Ideguchi K (2007). Factors associated with outcome of segmentectomy for non-small cell lung cancer: long-term follow-up study at a single institution in Japan. Lung Cancer.

[4] Okada M, Koike T, Higashiyama M (2006). Radical sublobar resection for small-sized non-small cell lung cancer: a multicenter study. J Thorac Cardiovasc Surg.

[5] Ginsberg RJ, Rubinstein LV (1995). Randomized trial of lobectomy versus limited resection for T1 N0 non-small cell lung cancer. Lung Cancer Study Group. Ann Thorac Surg.

[6] Ettinger DS, Wood DE, Aisner DL (2017). Non-Small Cell Lung Cancer, Version 5.2017, NCCN Clinical Practice Guidelines in Oncology. J Natl Compr Canc Netw.

[7] Rudin CM, Ismaila N, Hann CL (2015). Treatment of Small-Cell Lung Cancer: American Society of Clinical Oncology Endorsement of the American College of Chest Physicians Guideline. J Clin Oncol.

[8] Yendamuri S, Sharma R, Demmy M (2013). Temporal trends in outcomes following sublobar and lobar resections for small (< /= 2 cm) non-small cell lung cancers-a Surveillance Epidemiology End Results database analysis. J Surg Res.

[9] Keenan RJ, Landreneau RJ, Maley Jr RH (2004). Segmental resection spares pulmonary function in patients with stage Ⅰ lung cancer. Ann Thorac Surg.

[10] Schuchert MJ, Pettiford BL, Keeley S (2007). Anatomic segmentectomy in the treatment of stage Ⅰ non-small cell lung cancer. Ann Thorac Surg.

[11] Cao C, Chandrakumar D, Gupta S (2015). Could less be more?-A systematic review and meta-analysis of sublobar resections versus lobectomy for non-small cell lung cancer according to patient selection. Lung Cancer.

[12] Nakamura H, Kawasaki N, Taguchi M (2006). Survival impact of epidermal growth factor receptor overexpression in patients with non-small cell lung cancer: a *meta*-analysis. Thorax.

[13] Dominguez-Ventura A, Cassivi SD, Allen MS (2007). Lung cancer in octogenarians: factors affecting long-term survival following resection. Eur J Cardiothorac Surg.

[14] Liu T, Liu H, Li Y (2014). Early lung cancer in the elderly: sublobar resection provides equivalent long-term survival in comparison with lobectomy. Contemp Oncol (Pozn).

[15] De Zoysa MK, Hamed D, Routledge T (2012). Is limited pulmonary resection equivalent to lobectomy for surgical management of stage Ⅰ non-small-cell lung cancer?. Interact Cardiovasc Thorac Surg.

[16] Mery CM, Pappas AN, Bueno R (2005). Similar long-term survival of elderly patients with non-small cell lung cancer treated with lobectomy or wedge resection within the surveillance, epidemiology, and end results database. Chest.

[17] Razi SS, John MM, Sainathan S (2016). Sublobar resection is equivalent to lobectomy for T1a non-small cell lung cancer in the elderly: a Surveillance, Epidemiology, and End Results database analysis. J Surg Res.

[18] Kilic A, Schuchert MJ, Pettiford BL (2009). Anatomic segmentectomy for stage Ⅰ non-small cell lung cancer in the elderly. Ann Thorac Surg.

[19] Takizawa T, Haga M, Yagi N (1999). Pulmonary function after segmentectomy for small peripheral carcinoma of the lung. J Thorac Cardiovasc Surg.

[20] Okami J, Ito Y, Higashiyama M (2010). Sublobar resection provides an equivalent survival after lobectomy in elderly patients with early lung cancer. Ann Thorac Surg.

[21] Zhang J, Xue ZQ, Chu XY (2012). Surgical treatment and prognosis of octogenarians with non-small cell lung cancer. Asian Pac J Trop Med.

[22] Warwick R, Mediratta N, Shackcloth M (2013). Wedge resection verses lobectomy for stage 1 non-small-cell lung cancer. Asian Cardiovasc Thorac Ann.

[23] Lin L, Hu D, Zhong C (2013). Safety and efficacy of thoracoscopic wedge resection for elderly high-risk patients with stage Ⅰ peripheral non-small-cell lung cancer. J Cardiothorac Surg.

[24] Dell'Amore A, Monteverde M, Martucci N (2013). Early and long-term results of pulmonary resection for non-small-cell lung cancer in patients over 75 years of age: a multi-institutional study. Interact Cardiovasc Thorac Surg.

[25] Dell'Amore A, Monteverde M, Martucci N (2015). Lobar and sub-lobar lung resection in octogenarians with early stage non-small cell lung cancer: factors affecting surgical outcomes and long-term results. Gen Thorac Cardiovasc Surg.

[26] Wang J, Zhao H (2016). Issues need to be considered in sublobectomy for early stage lung cancer. Zhongguo Fei Ai Za Zhi.

[27] Yang F, Sui X, Chen X (2016). Sublobar resection versus lobectomy in surgical treatment of elderly patients with early-stage non-small cell lung cancer (STEPS): study protocol for a randomized controlled trial. Trials.

[28] Reveliotis K, Kalavrouziotis G, Skevis K (2014). Wedge resection and segmentectomy in patients with stage Ⅰ non-small cell lung carcinoma. Oncol Rev.

[29] Hou B, Deng XF, Zhou D (2016). Segmentectomy versus wedge resection for the treatment of high-risk operable patients with stage Ⅰ non-small cell lung cancer: a *meta*-analysis. Ther Adv Respir Dis.

[30] Smith CB, Swanson SJ, Mhango G (2013). Survival after segmentectomy and wedge resection in stage Ⅰ non-small-cell lung cancer. J Thorac Oncol.

[31] Schuchert MJ, Abbas G, Awais O (2012). Anatomic segmentectomy for the solitary pulmonary nodule and early-stage lung cancer. Ann Thorac Surg.

[32] Tsutani Y, Miyata Y, Nakayama H (2014). Appropriate sublobar resection choice for ground glass opacity-dominant clinical stage ⅠA lung adenocarcinoma: wedge resection or segmentectomy. Chest.

[33] Altorki NK, Kamel MK, Narula N (2016). Anatomical segmentectomy and wedge resections are associated with comparable outcomes for patients with small cT1N0 non-small cell lung cancer. J Thorac Oncol.

[34] Sienel W, Stremmel C, Kirschbaum A (2007). Frequency of local recurrence following segmentectomy of stage ⅠA non-small cell lung cancer is influenced by segment localisation and width of resection margins-implications for patient selection for segmentectomy. Eur J Cardiothorac Surg.

[35] Luzzi L, Marulli G, DE Palma A (2016). Prognostic factors and clinical outcome in patients with surgically resected peripheral N0 adenocarcinoma < 3 cm. Minerva Chir.

[36] Nomori H, Mori T, Ikeda K (2012). Segmentectomy for selected cT1N0M0 non-small cell lung cancer: a prospective study at a single institute. J Thorac Cardiovasc Surg.

[37] Okada M, Nishio W, Sakamoto T (2005). Effect of tumor size on prognosis in patients with non-small cell lung cancer: the role of segmentectomy as a type of lesser resection. J Thorac Cardiovasc Surg.

[38] Sugi K, Kobayashi S, Sudou M (2010). Long-term prognosis of video-assisted limited surgery for early lung cancer. Eur J Cardiothorac Surg.

[39] Qiu C, Wang G, Xu J (2017). Sublobectomy versus lobectomy for stage Ⅰ non-small cell lung cancer in the elderly. Int J Surg.

[40] Stiles BM, Kamel MK, Nasar A (2017). The importance of lymph node dissection accompanying wedge resection for clinical stage ⅠA lung cancerdagger. Eur J Cardiothorac Surg.

[41] El-Sherif A, Fernando HC, Santos R (2007). Margin and local recurrence after sublobar resection of non-small cell lung cancer. Ann Surg Oncol.

[42] Mohiuddin K, Haneuse S, Sofer T (2014). Relationship between margin distance and local recurrence among patients undergoing wedge resection for small (< /=2 cm) non-small cell lung cancer. J Thorac Cardiovasc Surg.

[43] Maurizi G, D'Andrilli A, Ciccone AM (2015). Margin distance does not influence recurrence and survival after wedge resection for lung cancer. Ann Thorac Surg.

